# Maternal and neonatal risk factors for neonatal respiratory distress syndrome in term neonates in Cyprus: a prospective case–control study

**DOI:** 10.1186/s13052-021-01086-5

**Published:** 2021-06-03

**Authors:** Paraskevi Stylianou-Riga, Theodora Boutsikou, Panayiotis Kouis, Paraskevi Kinni, Marina Krokou, Andriani Ioannou, Tania Siahanidou, Zoi Iliodromiti, Thalia Papadouri, Panayiotis K. Yiallouros, Nicoletta Iacovidou

**Affiliations:** 1 Neonatal Intensive Care Unit, “Archbishop Makarios III” Hospital, Nicosia, Cyprus; 2 Respiratory Physiology Laboratory, Medical School, University of Cyprus, 2029 Aglantzia, Nicosia, Cyprus; 3 Neonatal Department, Aretaieio Hospital, Medical School, National and Kapodistrian University of Athens, Athens, Greece; 4 Neonatal Unit, First Department of Pediatrics, ‘Aghia Sophia’ Children’s Hospital, Athens, Greece

**Keywords:** Neonatal respiratory distress syndrome, Maternal risk factors, Neonatal risk factors, Caesarean section

## Abstract

**Background:**

Neonatal respiratory distress syndrome (NRDS) is strongly associated with premature birth, but it can also affect term neonates. Unlike the extent of research in preterm neonates, risk factors associated with incidence and severity of NRDS in term neonates are not well studied. In this study, we examined the association of maternal and neonatal risk factors with the incidence and severity of NRDS in term neonates admitted to Neonatal Intensive Care Unit (NICU) in Cyprus.

**Methods:**

In a prospective, case-control design we recruited term neonates with NRDS and non-NRDS admitted to the NICU of Archbishop Makarios III hospital, the only neonatal tertiary centre in Cyprus, between April 2017–October 2018. Clinical data were obtained from patients’ files. We used univariate and multivariate logistic and linear regression models to analyse binary and continuous outcomes respectively.

**Results:**

During the 18-month study period, 134 term neonates admitted to NICU were recruited, 55 (41%) with NRDS diagnosis and 79 with non-NRDS as controls. In multivariate adjusted analysis, male gender (*OR*: 4.35, 95% CI: 1.03–18.39, *p* = 0.045) and elective caesarean section (*OR*: 11.92, 95% CI: 1.80–78.95, *p* = 0.01) were identified as independent predictors of NRDS. Among neonates with NRDS, early-onset infection tended to be associated with increased administration of surfactant (*β*:0.75, 95% CI: − 0.02-1.52, *p* = 0.055). Incidence of pulmonary hypertension or systemic hypotension were associated with longer duration of parenteral nutrition (pulmonary hypertension: 11Vs 5 days, *p* < 0.001, systemic hypotension: 7 Vs 4 days, *p* = 0.01) and higher rate of blood transfusion (pulmonary hypertension: 100% Vs 67%, *p* = 0.045, systemic hypotension: 85% Vs 55%, *p* = 0.013).

**Conclusions:**

This study highlights the role of elective caesarean section and male gender as independent risk factors for NRDS in term neonates. Certain therapeutic interventions are associated with complications during the course of disease. These findings can inform the development of evidence-based recommendations for improved perinatal care.

**Supplementary Information:**

The online version contains supplementary material available at 10.1186/s13052-021-01086-5.

## Background

Neonatal Respiratory Distress Syndrome (NRDS) is the main cause of neonatal respiratory failure and death [1], as well as admission in Neonatal Intensive Care Unit (NICU) [2]. NRDS is more frequent in preterm neonates [3], but it can affect term neonates as well. Nevertheless, the underlying aetiologies of NRDS in term neonates are different to those of NRDS in preterm neonates [4], so that NRDS in term neonates is frequently perceived as a distinct pathology [5].

Even in term neonates the frequency of NRDS has been linked to gestational age [6] and caesarean section [7], especially when performed before 39 weeks of gestation [8–11]. Other risk factors that have been associated with NRDS in term neonates include neonatal asphyxia, maternal or fetal infection, premature rupture of membranes and male gender [12]. However, most publications on the association of risk factors with NRDS relied on retrospective or administrative data [13–18] or did not focus on NRDS severity, management and resolution [19–22]. The few studies that assessed the association of maternal or neonatal risk factors with incidence but also the outcomes and severity of NRDS in term neonates were limited by studying a mixed population of both preterm and term neonates [23–25] or by retrospective data collection [26].

In Cyprus, a country of 875,000 inhabitants, known for the very high frequency of caesarean section deliveries (52.2% of all deliveries in 2018) [27], the annual incidence of NRDS in term and pre-term neonates is currently unknown. Furthermore, the frequency of other risk factors for NRDS and their association with incidence and severity of NRDS has never been studied in this setting.

The aim of this study was to investigate prospectively the frequency of NRDS in term neonates in Cyprus and examine the association between prenatal, perinatal and postnatal factors with incidence and severity of NRDS among this population. We also aimed to examine the association of several therapeutic interventions with severity and complications of NRDS.

## Methods

### Study location

The study was performed at the grade III-IV NICU (48 infant beds capacity) of Archbishop Makarios III (NAM III) hospital which serves the whole of Cyprus as the single national tertiary referral centre for all high-risk pregnancies and neonates requiring intensive care support.

### Study population and case-control selection

We obtained the total number of births in Cyprus for 2017 and 2018 from the Health Monitoring Unit of the Ministry of Health and the National Statistics Department, and the total number of neonates hospitalised in the NICU for the same period from the Unit’s records. Term neonates (gestational age ≥ 37 weeks) that were hospitalised in the NICU between April 2017 and October 2018 were prospectively recruited. Term neonates were defined as NRDS cases if they required mechanical ventilation and surfactant administration and fulfilled at least two of the following criteria: (a) tachypnea, (b) central cyanosis in room air, (c) expiratory grunting, (d) intercostals or jugular retractions and nasal flaring and (e) oxygen supplementation requirement during the first 2 days of life [5, 28]. Term neonates without NRDS that received standard neonatal nursing care were defined as controls. Neonates with known chromosomal abnormalities and congenital anatomical anomalies were excluded from the study.

### Ethics approval

All guardians of participating neonates provided written informed consent and the study was approved by the Cyprus National Bioethics Committee (EEBK EΠ 2017.01.22) and the Research Committee of the Cyprus Ministry of Health (Protocol approval: 0416/2017).

### Data collection

Maternal, anthropometric and medical data, were collected from the mothers’ medical notes. Maternal clinical data included pre-existing chronic conditions such as diabetes mellitus, thyroid gland disorders and heart disease as well as data on pregnancy complications, gestational diabetes, hypertensive disorders of pregnancy (eclampsia, preeclampsia), placental abnormalities, infections and mode of delivery. Mortality and neonatal clinical data were collected until discharge from the NICU. Neonatal clinical data included gestational age, respiratory distress diagnosis, requirement for neonatal resuscitation at the delivery room, meconium stained amniotic fluid, Apgar score, pH and base excess on admission, hypotension during the first 24 h of life, nutrition status, treatment received, NICU duration of hospitalization and respective complications. For neonates with NRDS, several other clinical parameters were also collected such as duration of mechanical ventilation, number of surfactant-replacement doses, NRDS complications (pneumothorax, pulmonary hypertension, hemodynamic instability), neonatal infection, antibiotic administration and nutrition management. Additionally, results of laboratory measurements (e.g. blood gases and blood glucose, lactic acid and creatinine levels) and brain and heart ultrasound findings were also collected.

### Statistical analysis

Continuous variables are presented as means and 95% confidence intervals (95% CI) or medians and interquartile range (IQR), while categorical variables are presented as counts and percentages. Two-way comparisons between continuous variables were carried out using t-test and Mann-Whitney test for normally and non-normally distributed variables, respectively. Categorical variables were compared with chi-square test. Univariate and multivariate logistic regression analysis was carried out to assess the association of different variables with NRDS and crude and adjusted Odds Ratios (OR) were reported with 95% CI. Parameters that yielded significant associationsin the univariate analysis were included in the multivariate analysis. For the assessment of the effect of clinical parameters on NRDS severity, univariate and multivariate linear regression analyses were carried out. In addition, separate analyses were performed for three different measures of NRDS severity: (a) duration of NICU hospitalisation (b) duration of mechanical ventilation and (c) number of surfactant doses administered. All statistical analyses were performed using STATA 12 (StataCorp, TX). P_value_ < 0.05 was set as the cut-off for statistical significance.

## Results

During years 2017 and 2018, there were 9229 and 9329 live births in Cyprus, respectively. In 2017, 662 neonates (245 term) were admitted to the tertiary referral NICU of Cyprus and in 2018, 661 (243 term). Among the term neonates admitted to the NICU, 22.0% were diagnosed with NRDS in 2017 and 23.5% in 2018. During the 18-month study period (April 2017–October 2018), a total of 134 term neonates were recruited, 55 with NRDS diagnosis and 79 non-NRDS neonates as controls (Table 1). The primary reasons for NICU admission for non-NRDS neonates were: jaundice requiring only phototherapy treatment (21,5%), low birth weight (5%), mild feeding difficulties (11.4%), transient tachypnea (39.3%), suspicion of early neonatal infection with negative subsequent blood culture (12.7%), mild perinatal stress (7.6%) and prolonged rupture of membranes (2.5%).

**Table 1:** Demographic, clinical and treatment characteristics of mothers and neonates

	NRDS (*n* = 55)	No NRDS (*n* = 79)	*p*_value_
*Categorical*			
Female (%)	14/55 (25.5)	37/78 (47.4)	0.01
Smoking during pregnancy (%)	1/48 (2.01)	5/75 (6.7)	0.250
Gestational diabetes (%)	7/47 (14.9)	11/74 (14.9)	0.997
Thrombophilia (%)	1/48 (2.1)	3/75 (4.0)	0.559
Caesarean section (%)	32/55 (58.2)	39/79 (49.4)	0.315
Elective Caesarean (%)	29/32 (90.1)	21/39 (53.9)	0.001
IUGR (%)	1/51 (2.0)	7/74 (9.5)	0.092
Chorio-amnionitis (%)	0/45 (0.0)	1/74 (1.4)	0.434
Oligo-hydramnios (%)	2/45 (4.4)	1/77 (1.3)	0.279
Preeclampsia (%)	1/50 (2.0)	3/76 (4.0)	0.542
Prenatal steroids (%)	5/49 (10.2)	9/76 (11.8)	0.777
Fetal distress (%)	16/49 (32.7)	13/67 (19.4)	0.104
Frequent OB visits (%)	46/53(86.8)	75/79(95.0)	0.097
Blood transfusion (any) (%)	40/55 (72.7)	15/79 (18.9%)	< 0.001
Resuscitation at birth (%)	24/52 (46.2)	14/63 (22.2)	0.007
Plasma transfusion (%)	38/55 (69.1)	12/79 (15.2%)	< 0.001
RBC transfusion (%)	14/55 (25.4)	4/79 (5.1%)	0.001
Platelets transfusion (%)	2/55 (3.6)	1/79 (1.3%)	0.362
Dopamine administration (%)	33/55 (60.0)	3/79 (3.8%)	< 0.001
Dobutamine administration (%)	17/55 (30.9)	1/79 (1.3%)	< 0.001
*Continuous*			
Gestational Age (weeks)	38.3 (37.96, 38.62)	38.6 (38.3–38.8)	0.100*
Birthweight (gr)	3146 (3035, 3256)	3160 (3044–3276)	0.866
Maternal Age (years)	31.1 (29.9, 32.3)	30.8 (29.6–31.9)	0.696
Maternal BMI (kg/m^2^)	23.8 (22.1, 25.4)	23.7 (22.4–25.1)	0.970*
pH	7.31 (7.28,7.34)	7.40 (7.37–7.42)	< 0.001
Base Excess	−6.72 (−7.78, −5.66)	−4.92 (− 5.58, − 4.26)	0.004
Parenteral nutrition (days)	8.24 (3.96, 12.52)	1.51 (1.02, 1.99)	< 0.001
Intravenous Antibiotics (days)	6.98 (6.38, 7.58)	5.86 (4.58, 7.15)	0.115
Apgar at 5 min	8.92 (8.6–9.3)	9.46 (9.2–9.7)	0.002*

*IUGR* Intrauterine Growth Restriction, *OB* Obstetrician, *RBC* Red blood cells, *BMI* Body mass Index

*Mann Whitney U Test for comparison of medians

NRDS and non-NRDS neonates had similar gestational age (38.3 weeks Vs 38.6 weeks, p_value_: 0.100), birthweight (3145.9 g Vs 3160 g, p_value_: 0.866) and maternal risk factors such as gestational diabetes (14.9% Vs 14.9%, p_value_: 0.997), preeclampsia (2% Vs 4%, p_value_: 0.542) and thrombophilia (2.1% Vs 4.0%, p_value_:0.559). However, in comparison to the non-NRDS controls, NRDS term neonates were more frequently males (74.5% Vs 53.6%, p_value_: 0.01), had a lower mean Apgar Score at 5 min (8.92 Vs 9.46, p_value_: 0.002) and were more frequently born by elective caesarean section (90.1% Vs 53.9%, p_value_:0.001). In addition, compared to non-NRDS controls, NRDS term neonates required neonatal resuscitation more frequently (46.2% Vs 22.2%, p_value_: 0.007), were characterised by lower pH (7.31 Vs 7.40, p_value_ < 0.001) and lower base excess (− 6.72 Vs − 4.92 p_value_: 0.004), Lastly, duration of parenteral nutrition was higher among NRDS compared to non-NRDS neonates (8.24 days Vs 1.51, p_value_ < 0.001), while there was a tendency for longer duration of intravenous antibiotics administration although the difference was not statistically significant (6.98 days Vs 5.86 days, p_value_: 0.115).

In univariate analysis, incidence of NRDS was significantly associated with male gender (OR: 2.64, 95% CI: 1.24–5.61, p_value_: 0.011), elective caesarean section (OR: 8.29, 95% CI: 2.16–31.81, p_value_: 0.002) and Apgar score at 5 min (OR: 0.64, 95% CI: 0.44–0.92, p_value_: 0.016). The significant association between male gender and elective caesarean section persisted after adjustment for other confounders in multivariate analysis. The adjusted OR for male gender was 4.35 (95% CI: 1.03–18.39, p_value_: 0.045) and the adjusted OR forelective caesarean section was 11.92 (95% CI: 1.80–78.95, p_value_: 0.010). The results of the univariate and multivariate analysis are presented in detail in Tables 2 and 3 respectively.

**Table 2:** Associations between risk factors and incidence of NRDS (univariate analysis)

	OR	95% CI	*p*_value_
*Categorical*			
Male gender	2.64	1.24–5.61	0.011
Smoking	0.29	0.03–2.63	0.276
Gestational diabetes	1.00	0.36–2.80	0.997
Thrombophilia	0.51	0.051–5.05	0.566
Caesarean section	1.42	0.71–2.86	0.315
Elective Caesarean section	8.29	2.16–31.81	0.002
IUGR	0.19	0.02–1.60	0.128
Oligo-hydramnios	3.53	0.31–40.13	0.308
Preeclampsia	0.49	0.50–4.91	0.549
Prenatal steroids	0.85	0.27–2.69	0.777
Fetal distress	2.01	0.86–4.71	0.107
Frequent Obstetrician visits	0.35	0.09–1.26	0.109
*Continuous*			
Gestational Age (weeks)	0.82	0.59–1.11	0.200
Birthweight (gr)	0.99	0.99–1.00	0.864
Maternal Age (years)	1.01	0.94–1.09	0.693
Maternal BMI	1.00	0.94–1.07	0.970
Apgar at 5 min	0.64	0.44–0.92	0.016
Lactate Acid (mmol/L)	1.03	0.90–1.18	0.633

*IUGR* Intrauterine Growth Restriction, *BMI* Body Mass Index

**Table 3:** Associations between risk factors and incidence of NRDS (multivariate analysis)

	OR	95% CI	*p*_value_
*Categorical*			
Male gender	4.35	1.03–18.39	0.045
Smoking	8.65	0.31–240.15	0.203
Elective Caesarean section	11.92	1.80–78.95	0.010
IUGR	0.14	0.007–2.41	0.175
Fetal distress	0.32	0.04–2.11	0.239
Frequent Obstetrician visits	1.13	0.15–8.84	0.905
*Continuous*			
Gestational age (weeks)	1.52	0.65–3.53	0.330
Apgar score at 5 min	0.65	0.29–1.44	0.286

*IUGR* Intrauterine Growth Restriction

The relationship between clinical parameters and severity of NRDS was evaluated using three different outcome measures (duration of NICU stay, duration of mechanical ventilation and number of surfactant doses administered). Duration ofNICU stay was found to be associated with late-onset infection (*β*: 5.91, 95% CI: 1.14–10.66, p_value_: 0.01) in the univariate analysis, but statistical significance was attenuated after adjustment for other factors, although the effect estimate and its direction were similar (*β*: 5.43, 95% CI: 1.40–12.27, p_value_: 0.114) (Table 4). In contrast, duration of mechanical ventilation was not affected by early-onset (*β*: 0.58, 95% CI: − 1.42-2.58, p_value_: 0.557) or late-onset (*β*: 0.06, 95% CI: − 2.54-2.67, p_value_: 0.960) infection (Table 5). When number of administered surfactant doses were examined as a measure of NRDS severity, a significant association with early-onset infection (*β*:0.98, 95% CI:0.50–1.46, p_value_ < 0.001) and pulmonary hypertension (*β:*1.14, 95% CI:0.52–1.77, p_value_:0.001) were identified in the univariate analysis. In multivariate analysis, the association with early-onset infection demonstrated a similar magnitude of effect but was marginally non-significant (*β:*0.75, 95% CI: − 0.02-1.52, p_value_:0.055), while the effect of pulmonary hypertension was markedly attenuated (*β:*0.47, 95% CI: − 0.63-1.56, p_value_:0.388) (Table 6).

**Table 4:** Associations between clinical variables and duration of NICU stay

	Univariate analysis			Multivariate analysis		
	*β*	95% CI	*p*_value_	*β*	95% CI	*p*_value_
Stained Amniotic Fluid	−1.92	−6.13 - 2.27	0.36	−2.37	−7.86 - 3.13	0.384
Early-onset infection	1.18	−2.25 - 4.63	0.4	1.18	−4.07 - 6.43	0.647
Delivery at tertiary center	−1.78	−5.43 - 1.85	0.32	−3.74	−9.72 - 2.10	0.199
Hypotension (first 24 h)	−1.38	−4.89 - 2.12	0.43	−3.43	−8.72 - 1.86	0.194
Hemoglobin	−0.02	−0.74 - 0.69	0.94	−0.36	−1.45 - 0.744	0.512
Fever	1.38	−7.33 - 10.10	0.751	5.96	−8.09 - 20.01	0.391
Hypothermia	0.31	−5.82 - 6.44	0.919	−0.06	− 8.48 - 8.35	0.988
Late-onset infection	5.91	1.14–10.66	0.01	5.43	−1.40 - 12.27	0.114
Pulmonary Hypertension	−0.09	−4.49 - 4.30	0.965	1.45	−6.41 - 9.31	0.708

**Table 5:** Associations between clinical variables and duration of mechanical ventilation

	Univariate analysis			Multivariate analysis		
	*β*	95% CI	*p*_value_	*β*	95% CI	*p*_value_
Stained Amniotic Fluid	14.39	2.68–26.09	0.017	1.32	−0.77 - 3.42	0.206
Early-onset infection	2.30	0.72–3.89	0.005	0.58	−1.42 - 2.58	0.557
Delivery at tertiary center	−2.64	−13.36 - 8.08	0.623	0.25	−1.98 - 2.48	0.820
Hypotension (first 24 h)	5.31	−4.66 - 15.27	0.290	0.68	−1.34 - 2.69	0.497
Hemoglobin	−0.28	−0.63 - 0.07	0.118	0.06	−0.36 - 0.48	0.773
Fever	−0.72	−5.07 - 3.62	0.739	1.64	−3.73 - 6.99	0.536
Hypothermia	−1.1	−18.93 - 16.73	0.902	−0.12	−3.34 - 3.09	0.938
Late-onset infection	−2.31	−18.63 - 14.02	0.777	0.06	−2.54 - 2.67	0.960
Pulmonary Hypertension	17.63	6.57–28.69	0.002	2.82	−6.65 - 9.15	0.747

**Table 6:** Associations between clinical variables and surfactant doses

	Univariate analysis			Multivariate analysis		
	*β*	95% CI	*p*_value_	*Β*	95% CI	*p*_value_
Stained Amniotic Fluid	0.061	−0.64 - 0.76	0.863	−0.23	−0.99 - 0.54	0.547
Early-onset infection	0.98	0.50–1.46	0.000	0.75	−0.02 - 1.52	0.055
Delivery at tertiary center	0.19	−0.42 - 0.81	0.529	0.19	−0.66 - 1.05	0.649
Hypotension (first 24 h)	0.52	−0.04 - 1.07	0.067	0.19	−0.54 - 0.93	0.595
Hemoglobin	−0.072	−0.19 - 0.04	0.212	0.07	−0.09 - 0.23	0.392
Fever	−0.04	−1.43 - 1.35	0.952	0.68	−1.27 - 2.63	0.477
Hypothermia	0.204	−0.80 - 1.21	0.686	0.05	−0.96 - 1.04	0.932
Late-onset infection	−0.06	−0.89 - 0.78	0.890	−0.36	−1.31 - 0.59	0.443
Pulmonary Hypertension	1.14	0.52–1.77	0.001	0.47	−0.63 - 1.56	0.388

NRDS complicated with pulmonary hypertension was associated with significantly higher duration of parenteral nutrition (11 Vs 5 days, p_value_ < 0.001) and more frequent need for blood transfusion (100% Vs 67%, p_value_: 0.045) when compared to NRDS without pulmonary hypertension. Similarly, in NRDS neonates, those with hypotension required parenteral nutrition for a significantly higher number of days (7 Vs 4 days, p_value_:0.010) and received blood transfusion more frequently (84.9% Vs 54.6%, p_value_:0.013) compared to those without hypotension. Between NRDS neonates with and without late-onset infection, no significant difference in the distribution of treatment modalities was observed (Supplementary Table 1).

## Discussion

In this prospective, case-control study, we report the incidence and clinical characteristics of NRDS in term neonates in Cyprus and evaluate the association of prenatal, perinatal and postnatal risk factors with the appearance and severity of this condition. The annual incidence of NRDS, among term neonates admitted to the NICU in Cyprus, ranged from 22.0% in 2017 to 23.5% in 2018 and it was more frequent among males and neonates born with an elective caesarean section. Early-onset infection was marginally associated with increased administration of surfactant, while hypotension and pulmonary hypertension were associated with longer duration of parenteral nutrition and higher rate of blood transfusions.

A positive association between male gender and NRDS was reported by Zhao D et al. [29]. The protective effect of female gender can be explained by the augmenting effect of estrogens on alveolar development and surfactant production [30]. The important role of estradiol and progesterone for fetal lung development has been reported to be mediated by an increase in vascular endothelial growth factor (VEGF) [31], which stimulates the proliferation and maturation of alveolar type II cells [32]. In animal studies, chronic androgen exposure in utero was found to delay surfactant production in male embryos [33], possibly through the epidermal growth factor (EGF-R) and transforming growth factor-beta (TGFβ-R) signaling pathways [34].

Previous studies have demonstrated that elective caesarean section, in the absence of labor signs, is associated with increased risk for NRDS [4, 20, 35]. Onset of spontaneous labor has been shown to lead to rapid clearance of fetal lung fluids and lung maturation [10], while higher gestational age is predictive of a favorable respiratory prognosis even in term neonates undergoing elective caesarean section [36–38].

Very few studies have examined the association of clinical parameters with severity indices or outcomes in NRDS and most of them were limited by small sample size and inconsistencies in the examined risk factors [12, 39, 40]. In our study, we found late-onset and early-onset infection to be associated with duration of NICU stay and duration of mechanical ventilation respectively in univariate analysis. It is known that mechanical ventilation is an independent risk factor for development of neonatal infection [41, 42]. However, it is possible that development of septic shock as a result of early or late-onset infection may require or prolong the need for mechanical ventilation [43, 44]. Univariate analysis also demonstrated that pulmonary hypertension was associated with both longer duration of mechanical ventilation and increased number of surfactant doses. Although this finding was attenuated in multivariate analysis, it is in line with previous reports. More specifically, the mainstay for pulmonary hypertension management includes optimal lung expansion and adequate oxygenation [45–47], while exogenous surfactant administration has been shown to significantly improve outcomes of pulmonary hypertension secondary to NRDS [45, 48]. In our ventilated neonates, we implemented modern modes of mechanical ventilation with synchronized intermittent positive pressure ventilation (SIPPV), which has been shown to be associated with a shorter overall duration of ventilation in term neonates as compared to intermittent mandatory ventilation [49]. Nevertheless, more sophisticated methods of mechanical ventilation such as volume targeted ventilation are increasingly being used and have been shown to further improve clinical outcomes by allowing finer control of ventilated tidal volume [50, 51]. Well controlled ventilation avoids the risk of volutrauma due to high tidal volume, reduces hypocarbia and risk of brain injury in case of frequent tidal volume fluctuations and avoids very low expired tidal volume that has been associated with atelectotrauma and hypercarbia [52]. Future use of volume targeted ventilation in our NICU, is expected to further improve patient outcomes.

This study demonstrated that NRDS neonates with early-onset infection required increased surfactant administration as compared to NRDS neonates without early-onset infection. An increased requirement for surfactant therapy for early onset pneumonia has been previously reported in late preterm and term neonates [53], while a slower response to surfactant therapy was found in specific types of infection such as group B streptococcal pneumonia [54]. The most likely mechanism explaining the requirement of additional exogenous surfactant in early onset infection is the impairment of endogenous surfactant synthesis or secretion of proteinases and other microbial components that degrade or inhibit surfactant-associated proteins. These components have been found to be excreted by a number of different respiratory pathogens such as *P. aeruginosa* [55], adenovirus and respiratory syncytial virus [56, 57] and Aspergillus fumigatus [58]. Nevertheless, to date, the overall effect of surfactant therapy on mortality and pulmonary complications in infants with bacterial pneumonia is not clear and further research is required [59].

Pulmonary hypertension as well as systemic hypotension in NRDS term neonates were also strongly associated with duration of parenteral nutrition. Neonates in mechanical ventilation often have increased nutritional requirements and meeting these requirementsis a challenging task [60]. Parenteral nutrition is a necessary life sustaining practice [61] and according to the European Consensus Guidelines on the Management of Respiratory Distress Syndrome, administration of parenteral nutrition should be initiated as soon as possible to reduce growth delay in neonates [62]. Nevertheless, other authors suggest that parenteral nutrition should only be initiated after clinical stabilization of the neonate [63]. In our study, blood transfusion was more frequent in NRDS neonates compared to non-NRDS neonates, especially when NRDS was further complicated by pulmonary hypertension and systemic hypotension. Red blood cells transfusion is often required to prevent the effects of anemia among NRDS neonates [64] but administration should always adhere to standing guidelines due to the increased risk of complications [65].

The major strengths of this study include the prospective recruitment of participants as well as the prospective data collection which was characterised by high data completeness. Furthermore, the study benefits from a well-defined study population as twins and neonates with congenital abnormalities were excluded a priori. Lastly, given that NAM III hospital NICU serves as the only tertiary referral centre in Cyprus, the study population was not restricted by maternal socioeconomic status and thus results are not affected by selection bias and can be generalised across the socioeconomic spectrum. However, this work is also characterised by some limitations. For ethical reasons, neonates (with or without NRDS) that died during NICU hospitalisation, were not included in the study and thus the study is limited to only morbidity outcomes. Nevertheless, during the study period, mortality in the NICU among term neonates was very low (1/245 in 2017 and 0/243 in 2018). Furthermore, we assessed only short-term clinical severity outcomes and did not address the association of maternal and neonatal risk factors with long-term complications. Lastly, our dataset did not include information on neonatal morbidity scoring systems such as the Clinical Risk Index for Babies (CRIB) [66] that has been previously suggested to predict NRDS severity [67] in neonates.

## Conclusions

Male gender and elective cesarean section are significant risk factors for NRDS among term neonates admitted to NICU. NRDS complicated with early-onset infection requires higher surfactant dose while hypotension and pulmonary hypertension are associated with higher duration of parenteral nutrition and higher rate of blood transfusion. To our knowledge, this is the first study to examine term NRDS population in Cyprus. In this respect, our results highlight the importance of specific risk factors in the development and severity of NRDS in term neonates and can be used to inform evidence-based NRDS management protocols in the NICU, develop strategic planning for obstetric management and hopefully set the basis for further epidemiological studies.

## Supplementary Information

**Additional file 1: Table S1.** Association of treatment modalities and NRDS complications.

## Data Availability

The datasets generated and/or analyzed during the current study are not publicly available due to the requirements of Ethics approval but are available from the corresponding author on reasonable request.

## Abbreviations

NRDS: Neonatal Respiratory Distress Syndrome
NICU: Neonatal Intensive Care Unit
OR: Odds Ratio
CI: Confidence interval
IQR: Interquartile range
VEGF: Vascular endothelial growth factor
EGF-R: epidermal growth factor
TGFβ-R: Transforming growth factor-beta
SIPPV: Synchronized intermittent positive pressure ventilation
CRIB: Clinical Risk Index for Babies
SNAP: Score for Neonatal Acute Physiology
