# Supplementary material for: Maternal and neonatal risk factors for neonatal respiratory distress syndrome in term neonates in Cyprus: a prospective case–control study
Source: Ital J Pediatr. 2021 Jun 3;47:129. doi: 10.1186/s13052-021-01086-5 (PMC8176707; doi:10.1186/s13052-021-01086-5)
Supplement: Supplementary file 1 — Additional file 1: Table S1. Association of treatment modalities and NRDS complications. [file 13052_2021_1086_MOESM1_ESM.docx]

**Supplementary Table 1:** Association of treatment modalities and NRDS complications

| **Treatment Parameters** | **NRDS**  **with late onset infection** | **NRDS**  **without late onset infection** | **p_value_** | **NRDS**  **with pulmonary hypertension** | **NRDS**  **without pulmonary hypertension** | **p_value_** | **NRDS**  **with hypotension** | **NRDS without hypotension** | **p_value_** |
| --- | --- | --- | --- | --- | --- | --- | --- | --- | --- |
| Umbilical catheter (%) | 6/8 (75%) | 39/44 (88.6%) | 0.299 | 8/9 (88.9%) | 40/46 (86.9%) | 0.874 | 31/33 (93.9%) | 17/22 (77.3%) | 0.069 |
| Parenteral Nutrition* (days) | 6 (4-11) | 5.5 (3.0-8.5) | 0.573 | 11 (11-13) | 5.0 (3.0-7.0) | <0.001 | 7.0  (4.0-11.0) | 4.0 (2.0-7.0) | 0.010 |
| Blood transfusion (any) (%) | 4/8 (50%) | 33/44 (75%) | 0.251 | 9/9 (100%) | 31/46 (67%) | 0.045 | 28/33 (84.9%) | 12/22 (54.6%) | 0.013 |
| Platelets transfusion (%) | 0/8 (0%) | 2/44 (4.5%) | 0.539 | 2/9 (22%) | 0/46 (0%) | 0.001 | 2/33 (6%) | 0/22 (0%) | 0.239 |
| Plasma transfusion (%) | 4/8 (50%) | 32/44 (72.7%) | 0.200 | 9/9 (100%) | 29/46 (63%) | 0.028 | 26/33 (84.9%) | 12/22 (54.6) | 0.057 |
| RBC transfusion | 1/8 (12.5%) | 10/44 (22.7%) | 0.515 | 6/9 (66.7%) | 8/46 (17.4%) | 0.002 | 12/33 (36.4%) | 2/22 (9.1%) | 0.023 |

* Median and Interquartile Range, NRDS: Neonatal Respiratory Distress Syndrome, RBC: Red blood cells
